# Direct Quantification of mRNA and miRNA from Cell Lysates Using Reverse Transcription Real Time PCR: A Multidimensional Analysis of the Performance of Reagents and Workflows

**DOI:** 10.1371/journal.pone.0072463

**Published:** 2013-09-05

**Authors:** Yoon Khei Ho, Wen Ting Xu, Heng Phon Too

**Affiliations:** 1 Department of Biochemistry, National University of Singapore, Singapore, Singapore; 2 Saw Swee Hock School of Public Health, National University of Singapore, Singapore, Singapore; 3 Molecular Engineering of Biological and Chemical System/Chemical Pharmaceutical Engineering, Singapore–Massachusetts Institute of Technology Alliance, Singapore, Singapore; 4 Bioprocessing Technology Institute, A*STAR (Agency for Science, Technology and Research), Singapore, Singapore; Philipps University, Germany

## Abstract

Substantial efforts have been devoted to *in vitro* testing of candidate chemotherapeutics by profiling transcriptional changes across the collection of NCI-60 cell-lines. A work-flow with reagents that enable the direct quantification of RNA of different molecular sizes simultaneously in the same sample without laborious total RNA isolation will invariably increase the throughput and accuracy of the study. MicroRNAs (miRNAs) are known to regulate most cellular functions, acting post-transcriptionally by repressing numerous eukaryotic mRNAs. Recent findings on the remarkable stability of miRNA prompted us to investigate the feasibility of quantifying the expression levels of both mRNA and miRNA directly from cell lysates (cell-to-Ct). Multidimensional analyses of the expressions of mRNA and miRNA across seven NCI-60 cell lines and multiple reagents were conducted to assess the performances of these reagents and workflows for cell-to-Ct measurements using reverse transcription-quantitative polymerase chain reaction (RT-qPCR). Quantification of RNA species using lysates prepared from an *in-house* and one of the commercial reagents demonstrated comparable performance to those prepared by the more laborious and conventional method of using guanidinium-phenol-chloroform. Additionally, miRNA was found to be highly stable in the cell lysates when incubated at room temperature for prolonged period of time and subjected to multiple freeze-thaw cycles. In summary, this study demonstrated significant differences in pre-analytical performance of a variety of commercially available reagents and described a cost-effective reagent useful for rapid, scalable, and high-throughput workflow for the detection of mRNA and miRNA from the same biological sample.

## Introduction

Developed in the late 1980 s, National Cancer Institute (NCI) human tumour cell line anti-cancer drug screen (NCI-60) is well recognized as an essential tool for drug discovery [1], [2]. These cell lines have been used to discover molecular-targeted anticancer drugs, by correlating the sensitivity of one or more drugs to gene expression profiles [1]. While gene expression profiling has been applied to elucidate the mechanisms underlying cytotoxicity [3], the emerging view is that integrative analysis of gene signatures in concert with miRNA signatures may provide deeper mechanistic insights [4], [5] and to unravel novel drug targets.

miRNAs is intimately involved in cancer biology [6]–[8]. It is well known that miRNAs regulate gene expression post-transcriptionally and it has been suggested that up to 60% of human genes are targeted by miRNAs [9]. miRNAs have been shown to down regulate the expressions of a large number of mRNA [10]. The expression profiles of mRNA and miRNA of NCI-60 cell-lines have recently been shown to correlate to drug sensitivity and resistance [4], [11]–[13]. Additionally, some studies have demonstrated clinical outcomes to be highly predictive by using gene expression models [2]. Hence, delineating the relationships of miRNA and mRNA expression levels can provide deeper insights into disease mechanisms and the discoveries of diagnostic biomarkers and novel therapeutic targets. Thus, establishing accurate profiles of mRNA and miRNA expression are critical.

Expression levels of mRNA and miRNA are quantified by various methods, including Northern blotting, oligonucleotide microarrays, sequencing and RT-qPCR [14]. The procedural requirement for large amount of RNA, low throughput and low sensitivity highly restrict the use of Northern blotting in gene profiling for drug screening [15], [16]. While microarrays allow high throughput expression profiling, the accuracy and reliability of this approach are still contentious [17]. Next generation sequencing technologies allows the profiling of the entire transcriptome, including small RNAs [18]. However, recent studies have reported biases in some sequencing approaches [19], raising the question of the reliability and accuracy in miRNA quantification. In contrast, quantitative PCR (qPCR) has gained prominence over other detection platforms due to its higher assay sensitivity, wider dynamic range and greater precision [17], [20]. With the recent availability of high-throughput and low sample consumption qPCR arrays, large numbers of studies can now be conducted [21].Thus, qPCR is debatably the most reliable, sensitive and flexible technology for quantification of mRNA and miRNA currently.

RT-qPCR involves total RNA purification steps, where a conventional method of total RNA isolation using guanidinium-phenol-chloroform is both laborious and time consuming. Therefore, a rapid and simplified alternative pre-analytical method that allows direct quantification of miRNAs/mRNAs, without the need to isolated RNA is highly desirable. Some commercially available cell lysis buffers are optimized for direct reverse transcription of mRNA after cell lysis and subsequent amplification by qPCR. Using these reagents, it is reasonable to propose that it should be possible to directly quantify miRNA from cell lysates as it is remarkably stable, in contrast to mRNA [22], [23]. Here, we evaluated the feasibility of adapting some of these commercial cell lysis buffers for the direct quantification of mRNA and miRNA from the cell lysates (cell-to-Ct), and compared to the commonly used guanidinium-phenol-chloroform method.

## Materials and Methods

### Cell culture and cell lysis

The NCI-60 cell lines from 7 tissues of origin- A549 (lung, ATCC CCL185), HCT116 (colon, ATCC CCL247, gift from Prof. Sim Kim Ping), OVCAR8 (ovarian, gift from Prof. Jean Paul Thiery [24]), 786–0 (renal, ATCC CRL1932, gift from Dr. Deng Lih Wen), M14 (melanoma, gift from Prof. Marie-Véronique Clément [25]), PC3 (prostate, ATCC CRL1435), U251 (central nervous system, ATCC 09063001) were grown in Dulbecco's minimum essential medium (DMEM) supplemented with 10% heat-inactivated fetal bovine serum (FBS, Hyclone, Logan, UT, USA), 100 U/mL penicillin and 100 μg/mL streptomycin (PS, Sigma). All cells were cultured in incubator with 5% CO2 humidified atmosphere at 37°C. The 7 cell lines in a 96-well plate (2×10^4^ cells per well, biological quadruplicates) were lysed with RealTime Ready Cell Lysis Kit (Roche), FastLane Cell cDNA Kit (Qiagen), Buffer of Cell Lysate for Reverse Transcription (Signosis BioSignal Capture), iScript™ RT-qPCR Sample Preparation Reagent (Bio-Rad Laboratories), or MicroRNA Cells-to-CT™ Kit (ABI Ambion) according to manufacturer recommendation. *In-house* cell lysis reagent [26], [27] was modified for detection of mRNA. This reagent contained 2% Triton X-100 and 2% NP40, 1/25 of RNAsecure (ABI) and 1 unit/µl of RQ1 DNAse (Promega, MA). Cells were washed once with PBS, and incubated with the *in-house* lysis buffer for 5 min at room temperature. The cell lysates were transferred to PCR strip tubes and incubated for 75°C for 5 min.

### Total RNA isolation

For comparison, the total RNA from the identical densities of cells was isolated using the guanidinium phenol reagent (TRIzol reagent; Invitrogen) in the presence of 20 μg/mL linear acrylamide (ABI), according to the manufacturer's instructions. The RNA pellet was dissolved in 50 µL of DEPC water.

### Reverse transcription (RT)

Reverse transcription was conducted immediately after cell lysis. Remaining cell lysates were stored in −20°C to examine the effect of repeated freezing and thawing on RNA stability. The cell lysates made up to 10% of the final RT mixture. Cell lysates, total RNA samples (8×10^2^ cells or 2 µL samples per RT reaction), and 10^8^ copies of synthetic miRNA (Sigma) were reverse transcribed with RT mixtures containing 100 U Improm II (Promega), 1× buffer, 1 mM dNTPs, 5 mM MgCl_2_, and 100 nM of each of the RT oligonucleotides (for RT of miRNA) or 10 µM random hexamer (for RT of mRNA) in a total volume of 20 μL for 60 min at 42°C. The reaction was terminated by heating at 75°C for 5 min.

### Selection of target genes

Target genes (mRNA and miRNA) were selected from profiling studies of NCI-60 using 41,000-probe Agilent Whole Human Genome Oligo Microarray and the 15,000-feature Agilent Human microRNA Microarray V2 [5]. These data sets are freely available in Cell Miner database (http://discover.nci.nih.gov/cellminer/). Microarray data includes technical duplicates of the selected cell lines was analyzed. The genes were sorted and grouped as high-, medium-, low-expressions based on microarray signal. Then, genes that were expressed at comparable levels across the seven cell lines were selected. Details on the selected genes and their expression level (indicated by Ct) validated by qPCR were provided in Table S1.

### Real-time PCR

The cDNA samples were then used for real-time PCR measurement with miRNA or mRNA-specific primers. Primers for mRNA were designed across specific exon boundaries (Beacon Designer 7). Mature miRNAs were detected with miR Φ Assays^TM^, A*STAR, Singapore. Primers for both RT and real-time PCR are listed in Table S1. Real-time PCR was performed on the CFX96 system (Bio-Rad) using SYBR Green I. PCR quantification (technical duplicates) of the mRNA and miRNA was performed as previously described [26], [28]. The threshold cycles (Ct) were calculated automatically using the CFX manager software (Bio-Rad). The efficiency of real-time RT-PCR was determined as previously described [29]. Briefly, 5-fold dilutions of cDNA of the isolated RNA controls were subjected to real-time RT-PCR. The standard curves of the mRNA and miRNA assays were obtained by plotting Ct versus Log (dilution factor). The assay efficiency was calculated by (10^1/S^–1)×100%, where S is the slope of the standard curve. This study is MIQE (Minimum Information for Publication of Quantitative Real-Time PCR Experiments) compliant.

### Statistical Analyses

The coefficient of variation (CV) was used to access the detection and extraction variability of the biological and technical replicates [28]. Where Student's *t*-test, was used, an unpaired two-tailed test was used, with the assumption that changes in gene expression is normally distributed [30].

## Results

### Lower extraction variability and comparable extraction efficiency of *in-house* cell-to-Ct reagent and iScript™ RT-qPCR sample preparation reagent (Bio-Rad) as compared to guanidinium-phenol-chloroform method

Direct detection of several RNA species from the same cell lysate without the need to isolate RNA dramatically increases the throughput and accuracy for integrative analysis of genetic networks. In order to identify suitable reagents for this purpose, the performances of an *in-house* and five commercially available reagents were compared to the guanidinium phenol reagent (Trizol). Comparisons were based on the variability (measured as coefficient of variation, CV) and efficiency of extraction (measured as Ct values) of mRNA and miRNA.

In this study, two sets of assays were designed (See Table S1). The first set of assays, consisting of designs to RPS2, VDAC1, hsa-miR-103, hsa-miR-532-3p, and hsa-miR-485-5p, was used to initially screen for the two best performing reagents for measuring mRNA and miRNA expressions. Then, a different set of assays for the detection of RPS8, ZNF106, RNF-167, hsa-miR-29a, hsa-miR-197, hsa-miR-297 was designed for further studies. All assays were MIQE compliant and the performances were assessed, showing high efficiency of amplification (>90%) with low intra-assay variation (See Table S2A). On the other hand, higher variation was observed with RT-qPCR, with CV ranging from 15.2% to 47.12% (See Table S2B). Next, the effect of lysis reagents on quantification was examined. All 7 cell-to-Ct reagents, when used at up to 10% of the final volume of the RT mixture, did not show any interference of the RT-qPCR process, indicative of the absence of inhibition (See Figure S1).

**Figure 1:**
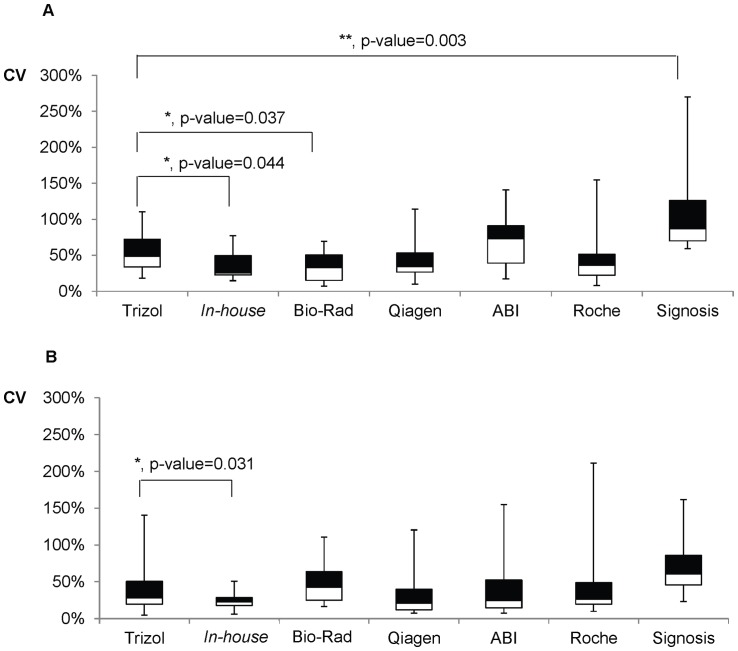
Extraction variability in mRNA/miRNA detection. Box plot representation of the coefficient of variation (CV) calculated from the Ct values for (A) mRNA (RPS2 and VDAC1) and (B) miRNA (hsa-miR-103, hsa-miR-532-3p, and hsa-miR-485-5p). Each box represented CVs of the combination of mRNAs/miRNAs detected from 7 cell lines. The 25th percentile to the 75th percentile (boxes), and ranges (whiskers) were shown. Significant differences in CVs between Trizol reagent and the cell-to-Ct reagents were calculated using the unpaired, two tailed student's t-test. *, p<0.05; **, p<0.005. Primer design, assay efficiency and intra- and inter-assay variations were reported in Table S1.

A comparison of the 7 commercial cell-to-Ct reagents for direct quantification and the use of Trizol reagent for the isolation of mRNAs/miRNAs from 2×10^4^ cells cultured in 96-well plates were first carried out. In order to verify the general utility of cell-to-Ct reagents for the NCI-60 panel, cell lines from seven tissues of origins were examined. Experiment was performed in biological quadruplicate (four RT reactions) and technical duplicate (two PCR reactions). For each cell line, a total of eight Ct values were obtained for each assay and CV was calculated. The pooled CVs of mRNA (Figure 1a) and miRNA (Figure 1b) assay detected from the 7 cell lines were presented. To examine the extraction variability of cell-to-Ct reagents, p value of the combined CVs of each cell-to-Ct reagent was calculated against CVs of Trizol reagent.

Overall, the extraction variability using Trizol reagent was comparable to the cell lysis buffers, except for the *in-house*, Bio-Rad and Signosis reagents. The *in-house* reagent exhibited lower extraction variability for both mRNA (p value 0.044) and miRNA (p value 0.031). It is worthy to note that the extraction variability of the *in-house* lysis buffer for miRNA exhibited low CV (<50%), which is within the range of the technical variability of RT-qPCR process (See Table S2B). The Bio-Rad reagent displayed superior consistency in extraction variability over Trizol reagent for mRNA isolation (p value 0.037). In contrast, the extraction variability of Signosis reagent for mRNA was significantly higher (p value 0.003) than Trizol reagent.

The extraction efficiency of the various cell-to-Ct reagents was compared to Trizol reagent, by evaluating the Ct values. It is important to note that the same cell number was used for extraction in all cases and the real time qPCR reactions of the various samples for each assay were conducted in the same assay plate so as to allow the direct comparison of extraction efficiency based on Ct values. We analyzed the Ct values of the aforementioned 5 candidate mRNA/miRNA (See Figure S2) and obtained the ΔCt (Ct of cell-to-Ct reagents–Ct of Trizol reagent) (Table 1). The extraction efficiencies of the *in-house* cell-to-Ct reagents were generally better or comparable to the use of Trizol across all cell lines and assays, with ΔCt ranging from −1.7 to 1.7 cycles. This was followed by the Signosis and Bio-Rad reagent, with ΔCt ±2 in most samples except for the detection of low expression VDAC1 in U251MG cell line when using both reagents and the detection of hsa-miR-532-3p with the Bio-Rad reagent.

**Table 1:** Extraction efficiency in the detection of mRNA and miRNA.^a^

Cell type	Gene	ΔCt (Ct of cell-to-Ct reagents – Ct of Trizol), average ± sem					
		In house	Bio-Rad	Qiagen	ABI	Roche	Signosis
**786–0**	RPS2	0.6±0.3	0.4±0.2	**2.5±0.2**	**2.6±0.5**	**2.3±0.1**	0.8±0.5
	VDAC1	−1.0±0.4	1.0±0.2	**4.0±0.3**	**7.6±0.6**	**2.8±0.3**	1.4±0.4
	hsa-miR-103	−1.4±0.0	1.4±0.2	**2.7±0.1**	0.8±0.1	**2.6±0.1**	0.3±0.6
	hsa-miR-532-3p	−1.7±0.2	1.5±0.1	**4.7±0.2**	1.3±0.1	**4.8±0.3**	0.4±0.6
	hsa-miR-485-5p	−1.7±0.5	−0.9±0.9	−0.2±0.1	−0.5±0.3	0.1±0.4	0.2±0.4
**A-549**	RPS2	0.1±0.4	−0.5±0.1	1.1±0.3	1.8±0.3	**2.2±0.1**	−0.7±0.5
	VDAC1	0.2±0.4	1.1±0.3	**2.4±0.2**	**7.0±0.2**	**4.0±0.3**	**2.6±0.5**
	hsa-miR-103	−0.2±0.2	0.4±0.1	**2.5±0.1**	1.0±0.1	**2.2±0.1**	1.2±0.4
	hsa-miR-532-3p	0.0±0.2	−0.4±0.3	**3.5±0.2**	1.2±0.3	**3.8±0.2**	1.0±0.4
	hsa-miR-485-5p	0.5±0.2	1.9±0.3	**2.3±0.3**	2.0±0.3	**2.2±0.3**	0.7±0.2
**HCT116**	RPS2	−0.7±0.1	−1.8±0.1	−1.5±0.1	1.1±0.2	0.7±0.2	−1.5±0.4
	VDAC1	−0.7±0.5	−1.6±0.3	0.7±0.2	**6.8±0.5**	2.0±0.3	−0.8±0.4
	hsa-miR-103	−1.3±0.2	−0.4±0.2	1.8±0.2	1.7±0.1	1.4±0.1	−0.2±0.4
	hsa-miR-532-3p	−0.9±0.2	−0.3±0.1	**3.4±0.3**	**2.7±0.3**	**3.5±0.1**	0.3±0.4
	hsa-miR-485-5p	−0.7±0.1	0.8±0.5	0.0±0.4	0.6±0.5	0.3±0.3	0.0±0.3
**M14**	RPS2	0.6±0.1	−1.1±0.1	−0.1±0.2	0.6±0.5	**2.8±0.2**	−0.9±0.7
	VDAC1	0.7±0.1	0.8±0.3	**2.3±0.3**	**6.7±0.5**	**3.8±0.3**	1.9±0.6
	hsa-miR-103	−0.6±0.1	0.2±0.3	**2.9±0.1**	1.1±0.1	**3.1±0.1**	1.3±0.2
	hsa-miR-532-3p	0.0±0.1	0.5±0.3	**3.6±0.1**	1.3±0.1	**4.3±0.2**	1.4±0.2
	hsa-miR-485-5p	−1.3±0.1	1.5±0.4	0.4±0.3	−0.1±0.4	0.6±0.3	0.6±0.3
**OVCAR8**	RPS2	0.4±0.2	−1.1±0.3	−0.1±0.1	1.3±0.4	1.8±0.1	−0.4±0.5
	VDAC1	−1.5±0.3	−0.9±0.3	−1.3±0.4	**6.2±0.4**	**4.6±0.7**	−1.3±0.9
	hsa-miR-103	−0.8±0.2	−0.4±0.1	**2.4±0.1**	0.6±0.1	**2.3±0.2**	1.0±0.5
	hsa-miR-532-3p	−0.9±0.2	**−2.2±0.5**	**2.9±0.4**	1.1±0.1	**3.3±0.3**	1.1±0.4
	hsa-miR-485-5p	−0.6±0.3	−0.1±0.2	0.2±0.6	0.9±0.7	1.0±0.9	0.8±0.7
**PC3**	RPS2	1.7±0.1	−0.1±0.1	1.5±0.1	0.9±0.1	**2.2±0.2**	0.0±0.5
	VDAC1	0.4±0.2	0.5±0.4	1.5±0.2	**6.9±0.4**	2.0±0.1	1.6±0.6
	hsa-miR-103	−0.5±0.1	0.9±0.2	**2.5±0.1**	1.4±0.2	**2.3±0.1**	1.1±0.3
	hsa-miR-532-3p	−0.3±0.2	−1.2±0.1	**3.7±0.1**	1.6±0.2	**4.2±0.2**	1.2±0.3
	hsa-miR-485-5p	−0.4±0.1	0.6±0.5	−0.9±0.2	−0.8±0.3	−1.4±0.3	−1.2±0.2
**U251MG**	RPS2	1.0±0.2	−0.8±0.1	−0.2±0.2	0.9±0.4	**3.2±0.3**	1.5±1.1
	VDAC1	−0.3±0.1	**2.8±0.3**	1.4±0.3	**6.6±0.6**	**4.2±0.2**	**3.1±0.6**
	hsa-miR-103	−0.4±0.1	0.8±0.4	**2.4±0.5**	−0.6±0.1	**2.4±0.8**	1.0±0.6
	hsa-miR-532-3p	−0.9±0.1	0.3±0.3	**3.9±1.6**	1.2±0.1	**4.2±0.3**	0.7±0.6
	hsa-miR-485-5p	0.5±0.1	1.5±0.2	0.5±0.1	**−2.5±0.1**	−1.2±0.1	−0.6±0.2

a The detected Ct values of the miRNA and mRNA assays from samples prepared with cell-to-Ct reagents were compared against Trizol reagent. The average ΔCt (Ct of cell-to-Ct reagents – Ct of Trizol) ± S.E.M. of the biological quadruplicates are summarized in the table. The values in bold represents average ΔCt >2 cycles.

On the other hand, the differences of Ct values between the Trizol reagent and some cell-to-Ct reagents were clearly evident. The ΔCt of the candidate genes in samples prepared with the ABI, Qiagen, and Roche reagents ranged from −2.5 to 7.6 cycles, −1.5 to 4.7 cycles, and −1.4 to 4.8 cycles, respectively. Although mRNA was measurable in samples lysed with the ABI reagent (MicroRNA Cells-to-CT™ Kit), the detection of low-expression VDAC1 was poor with ΔCt ranging from 6.2 to 7.6 cycles, suggesting the possibility of mRNA degradation during sample preparation. However, extraction of miRNA using the ABI reagent was equally efficient when compared to the use of Trizol reagent. The extraction efficiencies of the Qiagen and Roche reagents were poorer than that of the Trizol reagent in most cases. In an attempt to increase the extraction efficiency, the effect of an additional step to triturate the cells after incubation with the cell lysis buffer was examined. Ct values obtained in both cell models,786–0 and M14 cell lines, showed that trituration of cell layer did not improve the extraction efficiency (See Figure S3), indicating that the lower efficiencies achieved with Qiagen and Roche reagents were likely not to be due to physical limitations e.g, disruption of cells.

**Figure 2:**
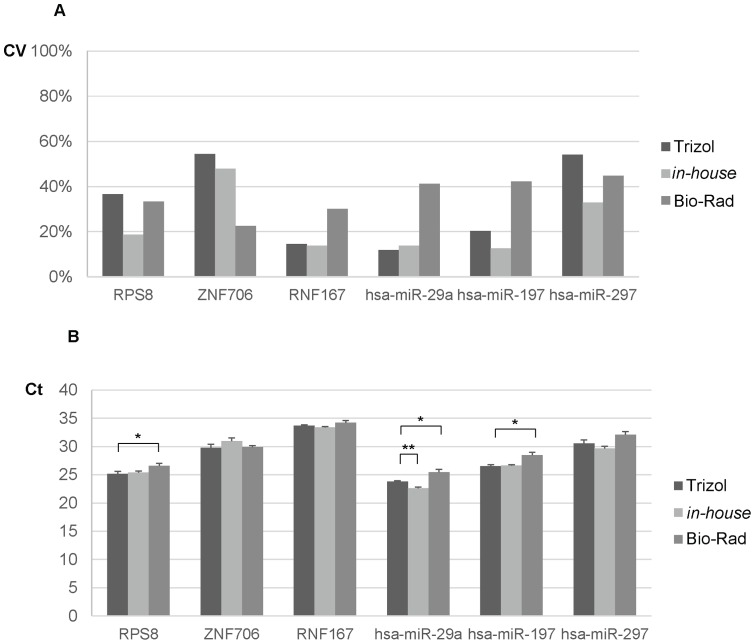
Validation of extraction variability and efficiency. M14 cell line (10^4^ cells) was cultured and lysed with the Trizol reagent, in-house and Bio-Rad cell-to-Ct reagents. After reverse transcription, the cDNA samples were amplified by RPS8, ZNF706, RNF167, hsa-miR-29a, hsa-miR-197, hsa-miR-297 PCR assays. Graph bars represent (A) CVs and (B) detected Ct values of the biological quadruplicates. The error bars referred to the S.E.M and the significant differences in CVs between the Trizol reagent and cell-to-Ct reagents were calculated using unpaired, two tailed student's t-test. *, p<0.05; **, p<0.005.

In general, the *in-house* and Bio-Rad cell-to-Ct reagent demonstrated equal if not better extraction efficiency and lower extraction variability in comparison to the Trizol reagent. Next, to further confirm the efficient recovery of RNA from the *in-house* and Bio-Rad reagent, similar experimental design was carried out with M14 cell line. Here, M14 cells were lysed and mRNA and miRNA quantification was conducted with the second set of assays. Both the *in-house* (CV 12.7%–47.8%) and Bio-Rad (CV 22.5%–44.7%) reagents showed comparable extraction efficiencies to the Trizol reagent (CV 11.9%–54.7%) (Figure 2A). Additionally, both cell-to-Ct reagents extract RNA equally well in comparison with the Trizol reagent (Figure 2B). The observed small differences in the Ct values of the three reagents were likely to be due to low technical variability of the real-time qPCR, ranging from 0.46–1.44 cycle.

To examine the possibility that the performances of the cell lysis buffers were dependent on the type of reverse transcription system used, RNA isolated with Trizol, *in-house* and Bio-Rad reagent were reverse transcribed using kits from Promega, Sigma and Enzymatics. Comparable Ct values were obtained using cDNA prepared by all three kits, suggesting that the performance of various lysis conditions is independent of the reverse transcription system used (See Figure S4). Collectively, this study showed that these two RNA species (mRNA and miRNA) were extracted with high efficiencies and low variabilities using the *in-house* and Bio-Rad cell-to-Ct reagents.

### Linearity and detection limit

**Figure 3:**
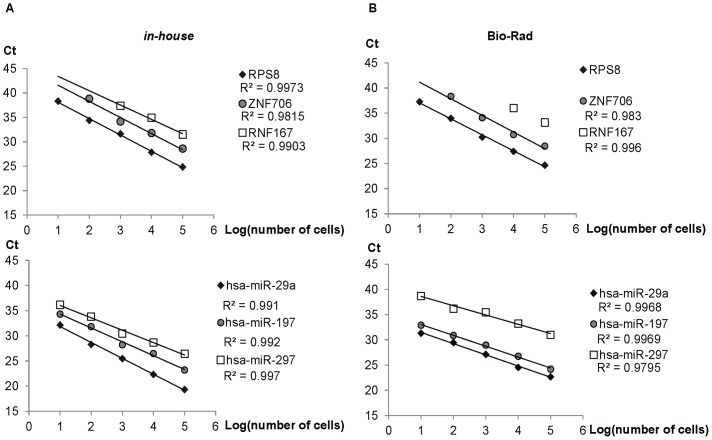
Excellent linearity of RNA detection. M14 cell line cultured in 96-wells at various densities (10, 100, 1000, 10,000 and 100,000 cells per well) were lysed directly by (A) in-house or (B) Bio-Rad lysis buffer. The cDNA samples generated from cell lysates were amplified by PCR assays from set 2. Standard curves for isolated RNAs were plotted as Ct versus Log (cells per RT). The experiments were conducted with biological duplicates.

We next investigated the performance of the *in-house* and Bio-Rad cell-to-Ct reagents by RT-qPCR in measuring mRNA and miRNA from 2×10 to 2×10^5^ M14 cells in 96-wells, with biological duplicates for each cell density. The excellent linearity in measuring mRNA (R^2^ 0.9815–0.9973) and miRNA (R^2^ 0.795–0.997) directly from cell lysates prepared with the *in-house* (Figure 3A) and Bio-Rad lysis buffers (Figure 3B) suggested that both RNA species can be reliably quantified from as few as 2×10 cells. Nevertheless, the medium-abundance gene, ZNF706 was measureable from at least 2×10^2^ cells per well. Expectedly, the direct quantification of the low-abundance mRNA (RNF167) was less sensitive with lysates prepared from cultures containing lesser than 2×10^3^ and 2×10^2^ cells per well with the *in-house* and Bio-Rad lysis buffer, respectively.

### miRNA but not mRNA is stable in cell lysate

**Figure 4:**
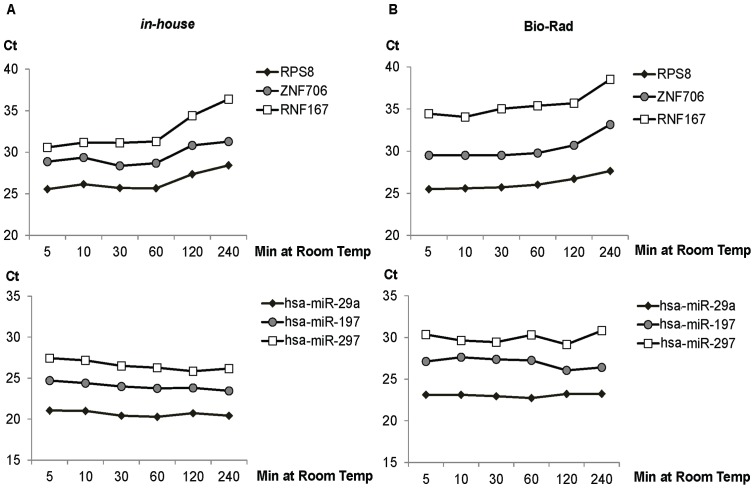
Remarkable stability of miRNA but not mRNA at room temperature. M14 cultured in 96-wells at 10^4^ cells per well (biological triplicates) were lysed directly by (A) in-house or (B) Bio-Rad lysis buffers and incubated at room temperature across various durations (5, 10, 30, 60, 120, 240 min). The cDNA samples generated from cell lysates were amplified by PCR assays from set 2. Ct was plotted against duration of incubation to examine the stability of RNA in room temperature. The experiments were conducted with biological duplicates.

It is well known that the miRNA/mRNA expression profiles largely depends on the quality of the input RNA material [31]. It has been previously shown that RNA degradation, profoundly affects the accuracy of mRNA [32], [33] and miRNA [34] quantification. This prompted us to further investigate the stability of these two different species of RNA in the cell lysates at room temperature. M14 cell lysates (2×10^4^ cells in biological duplicates) were incubated at room temperature for various durations following cell lysis, then reverse transcribed and quantified. Remarkable stability of mature miRNAs but not mRNA was observed in lysates prepared with the *in-house* and Bio-Rad lysis buffer (Figure 4). Significant decrease in the amounts of quantifiable mRNA was observed with samples prepared with the *in-house* and Bio-Rad reagents after prolonged incubation of more than 60 min (Figure 4A).

### Remarkable stability of mRNA in the *in*-*house* lysis reagent but not Bio-Rad lysis reagent with multiple freeze thaw cycles

**Figure 5:**
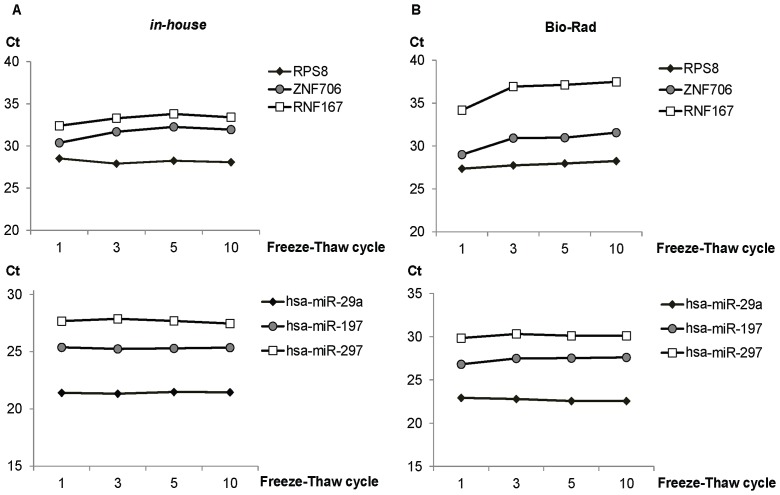
Remarkable stability of mRNA and miRNA at repeated freeze-thaw cycles in the *in-house* lysates. M14 cultured in 96-wells at 10^4^ cells per well (biological triplicates) were directly lysed by (A) in-house or (B) Bio-Rad lysis buffers. Cell lysates were collected and aliquoted. The lysates were then frozen at −70°C, thawed in a temperature-controlled water bath at 25°C, and the freeze-thaw cycles repeated. The miRNA and mRNA expressions were quantified following 1, 3, 5, 10 freeze-thaw cycles. The qPCR assays from set 2 were used for quantifications. Ct was plotted against freeze-thaw cycles to examine the stability of RNA. The experiments were conducted in biological duplicates.

The effect of freeze-thaw was next evaluated using M14 cell lysates (2×10^4^ cells, biological duplicates) subjected to one (control), three, five, ten freeze-thaw cycles. While miRNA stability was not affected by repeated freeze-thaw, changes in the levels of mRNA after three freeze-thaw cycles were observed with Bio-Rad but not *in-house* lysis buffer (Figure 5). It appeared that selective mRNAs were affected differently in samples using Bio-Rad cell-to-Ct reagent (Figure 5B), possibly by degradation, an observation consistent with previous findings [22], [35].

## Discussion

With the increased interest in the integrative analysis of the expression profiles of miRNA and mRNA for drug discovery using human cell lines [2], [5], [11], [12], rapid, scalable, and cost-effective methods for simultaneous quantification of the two RNA species are highly desirable. The direct cell lysis to RT-qPCR detection method described herein is simple to implement, showed excellent performance, and provided flexibility for high-throughput quantification of mRNA and miRNAs.

Integrative analysis of miRNA and mRNA profiles is gaining popularity because miRNAs, as the master regulators in gene expression networks can influence many biological processes. Gene expression profiling is known to be influenced not only by the robustness of the quantification methods, but also by various pre-analytical issues including quality of the input RNA material [32], [33], [36]–[38]. Phenol-chloroform extraction is the most widely used method for the isolation of total RNAs, including small RNA [23]. The major drawbacks with this method include the use of highly toxic reagents and the processes are time consuming and labor intensive which may result in increased risk of cross contamination [39], especially when large number of samples are prepared.

To date, most of the commercially available cell lysis buffers or cell-to-Ct reagents are designed for direct quantification of mRNA or microRNA without the need for RNA isolation. In this study, both Bio-Rad and an *in-house* reagents displayed comparable performance to the widely-used reagent, Trizol.Although some commercially available cell-to-Ct reagents were not specifically designed for miRNAs measurements, we were able to quantify miRNA with high sensitivity and reproducibly from cell lysates directly. In contrast, the performance of ABI reagent, which was specifically designed for miRNA quantification, was rather poor when measuring mRNA.

The rationale behind the formulation of the *in-house* cell lysis reagent was that cells were lyzed by mild non-ionic detergents (NP-40, Triton X-100) in the presence of RNAse inhibitors (RNAsecure) and the genomic DNA digested with RQ1 DNAse. This cost-effective reagent out performed all the commercially available reagents tested in quantifying miRNA and mRNA, even at low cell density. With the cell types examined, miRNA in the lysates were stable for a prolong period of time at room temperature (up to 3 h). It has been reported that the stability of miRNAs are generally more robust, enabling the accurate measurements even in degraded RNA samples where reliable mRNA analyses are not possible [22]. Similarly, accurate measurements of miRNA but not mRNA is possible with RNA extracted from formalin-fixed, paraffin embedded (FFPE) tissue [40]. Unexpectedly, the mRNA in the lysates prepared using *in-house* reagent but not Bio-Rad cell-to-Ct reagent were stable with multiple freeze-thaw cycle. In addition, the causes for mRNA degradation such as prolonged incubation at room temperature (>60 minutes) and multiple freeze thaw cycles (>3 cycles) were identified in this study (Figure 4 and 5). Thus, measures can be taken to minimize mRNA degradation. Taken together, simultaneous quantification of miRNA and mRNA with *in-house* and Biorad reagents is highly feasible.

## Supporting Information

Figure S1 **RT-qPCR efficiencies of the assays were not affected by the compositions present in lysis buffers.** GFP RNA, 10^10^ copies/µL was diluted to 10^7^, 10^6^, 10^5^, 10^4^, 10^3^, and 10^2^ copies/µL with DEPC water or various lysis buffers. The GFP RNA samples were diluted 10× in the RT mixtures and reverse transcribed. The cDNA were amplified by RT-qPCR assay. PCR efficiencies were then calculated from standard curves plotted as Ct versus Log.
(TIF)

Figure S2 **Detected Ct values of PCR assays.** Box plot representation of the Ct values of various PCR assays amplified in cDNA samples of (A) 786–0, (B) A549, (C) HCT-116, (D) M14, (E) OVCAR8, (F) PC3, and (G) U251MG cell lines. Significant differences in Cts between Trizol reagent and the cell-to-Ct reagents were calculated using the unpaired, two tailed student's t-test. *, p<0.01; **, p<0.001.
(TIF)

Figure S3 **Detection of miRNAs was not improved by trituration of cell layer during lysis step.** M14 and 786–0 cells cultured in 96-wells at 10^4^ cells per well (biological triplicates) were directly lysed by (A) Qiagen or (B) Roche lysis buffers with or without an additional step of triturate. The cDNA samples were generated and amplified by RT-qPCR. Graph bars represent detected Ct values and the error bars refer to the S.E.M of 3 biological samples. Significant differences in the Ct values were calculated using the two tailed student's t-test. *, p<0.01.
(TIF)

Figure S4 **The performances of cell-to-Ct reagents were not dependent on the choice of reverse transcription systems.** U251MG cultured in 96-wells at 10^4^ cells per well were directly lysed with Trizol reagent, *in-house* and Biorad cell-to-Ct reagents. Total RNA samples or cell lysates were then reversed transcribed with various reverse transcription kits (ImProm-II™ Reverse Transcription System, Promega; Enhanced Avian HS RT-PCR Kit, Sigma; M-MuLV Reverse Transcriptase, Enzymatics) according to the manufacturer's instructions. The cDNA samples were amplified and the Ct values determined. Graph bars represented Ct values and the error bars referred to the S.E.M of 3 biological samples. Significant differences in the Ct values between Trizol reagent and cell-to-Ct reagents were calculated using the two tailed student's t-test. *, p<0.01.
(TIF)

Table S1 **RT-qPCR assay design and performance.** Target genes were selected from Agilent Whole Human Genome Oligo Microarray and Agilent Human microRNA Microarray V2 data based on the microarray signals. Specific primers for mRNA were designed using Beacon designer. For microRNA detection, miRXES microRNA assays were used. The performance of these assays was validated by testing serial dilutions of cDNA from 786–0 cell line. Efficiencies of amplification were quantified. Average Ct/unit input of RNA, 500ng detected from the total RNA samples of the 8 selected cell lines was reported.
(TIF)

Table S2 **Intra assay variation and RT-qPCR variation.** (A) Total RNA sample isolated from M14 cells were reversed transcribed. Five replicates of the cDNA sample were amplified with various PCR assays. (B) Five RT reactions were prepared from the RNA sample isolated from M14 cells. The cDNA samples were then amplified by various PCR assays. Table summarizes the average Ct value ± S.D. and CVs of the assays used to measure the selected mRNAs and miRNAs in this study.
(TIF)
