# Supplementary figures and images for: Direct Quantification of mRNA and miRNA from Cell Lysates Using Reverse Transcription Real Time PCR: A Multidimensional Analysis of the Performance of Reagents and Workflows
Source: PLoS One. 2013 Sep 5;8(9):e72463. doi: 10.1371/journal.pone.0072463 (PMC3764000; doi:10.1371/journal.pone.0072463)

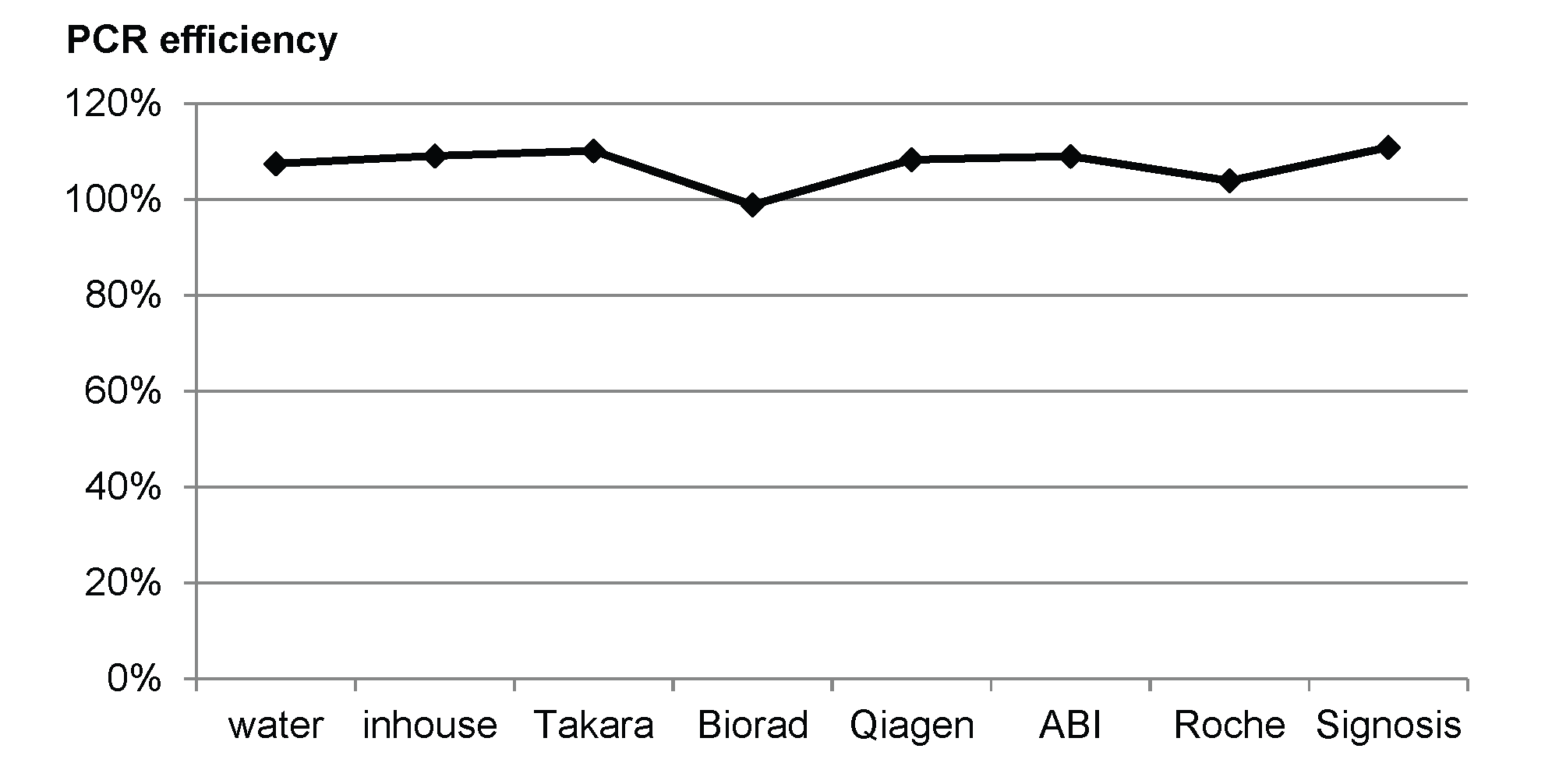

Supplement: Figure S1 — RT-qPCR efficiencies of the assays were not affected by the compositions present in lysis buffers. GFP RNA, 1010 copies/µL was diluted to 107, 106, 105, 104, 103, and 102 copies/µL with DEPC water or various lysis buffers. The GFP RNA samples were diluted 10× in the RT mixtures and reverse transcribed. The cDNA were amplified by RT-qPCR assay. PCR efficiencies were then calculated from standard curves plotted as Ct versus Log. (TIF) [file pone.0072463.s001.tif]

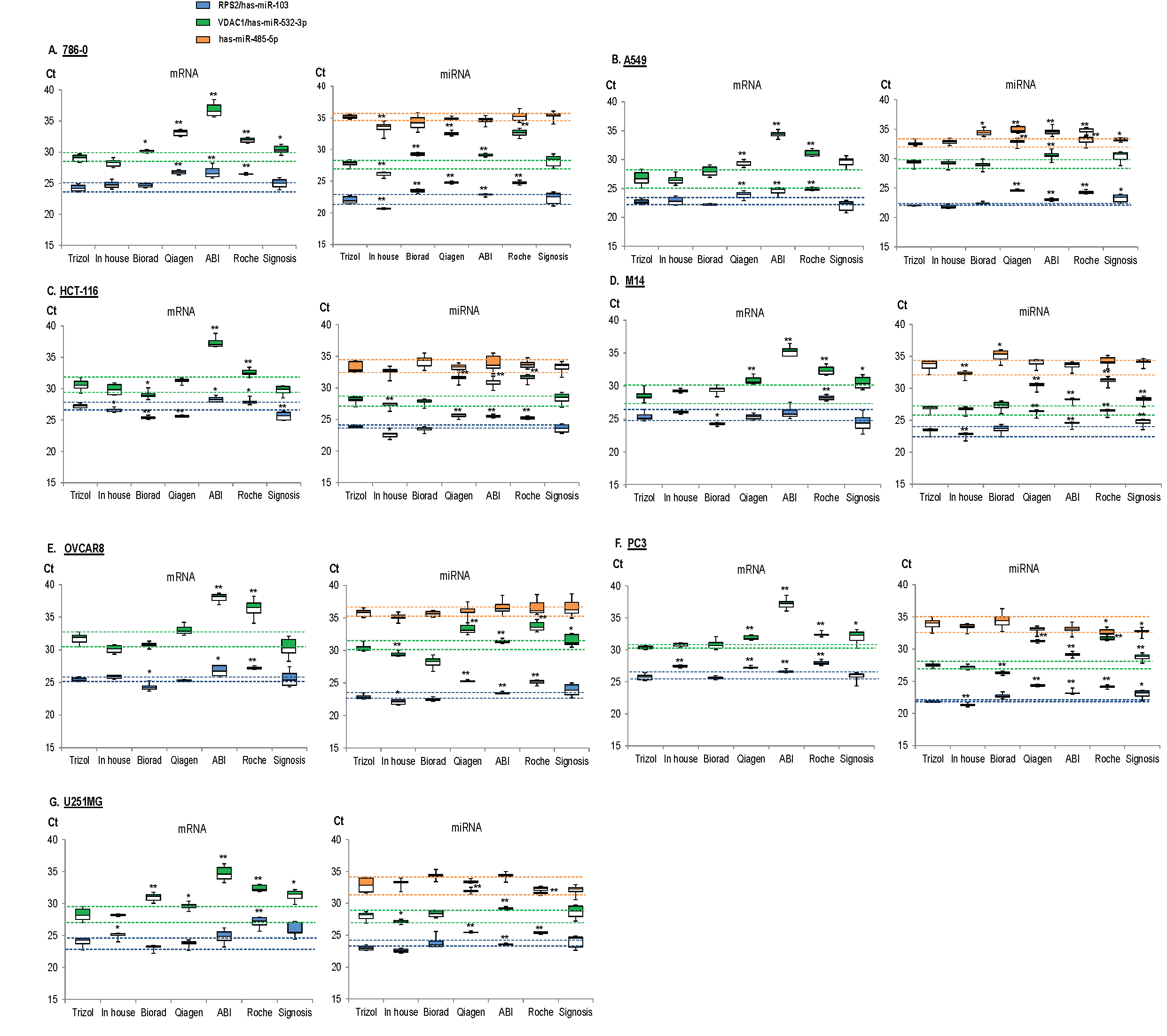

Supplement: Figure S2 — Detected Ct values of PCR assays. Box plot representation of the Ct values of various PCR assays amplified in cDNA samples of (A) 786–0, (B) A549, (C) HCT-116, (D) M14, (E) OVCAR8, (F) PC3, and (G) U251MG cell lines. Significant differences in Cts between Trizol reagent and the cell-to-Ct reagents were calculated using the unpaired, two tailed student's t-test. *, p<0.01; **, p<0.001. (TIF) [file pone.0072463.s002.tif]

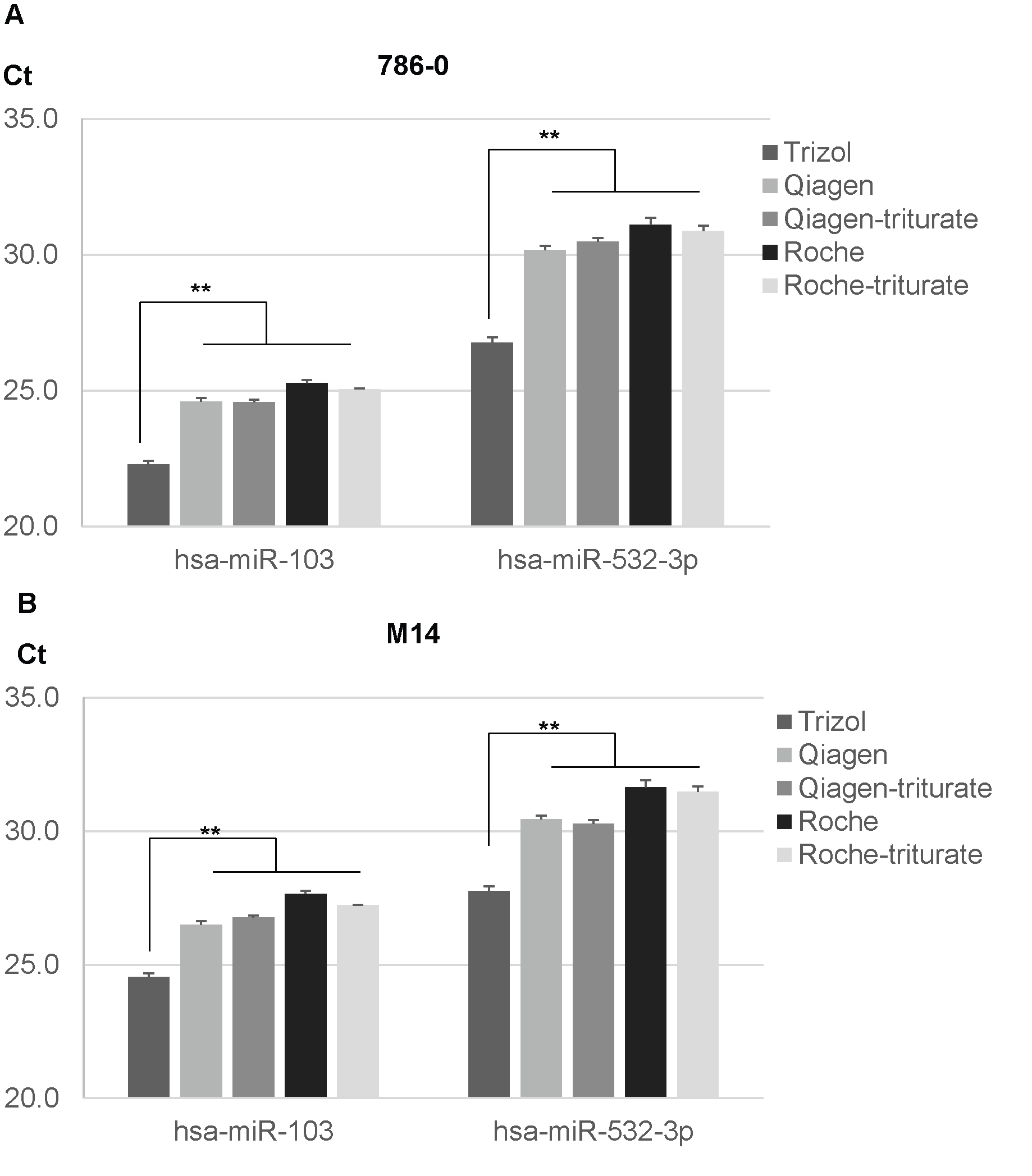

Supplement: Figure S3 — Detection of miRNAs was not improved by trituration of cell layer during lysis step. M14 and 786–0 cells cultured in 96-wells at 104 cells per well (biological triplicates) were directly lysed by (A) Qiagen or (B) Roche lysis buffers with or without an additional step of triturate. The cDNA samples were generated and amplified by RT-qPCR. Graph bars represent detected Ct values and the error bars refer to the S.E.M of 3 biological samples. Significant differences in the Ct values were calculated using the two tailed student's t-test. *, p<0.01. (TIF) [file pone.0072463.s003.tif]

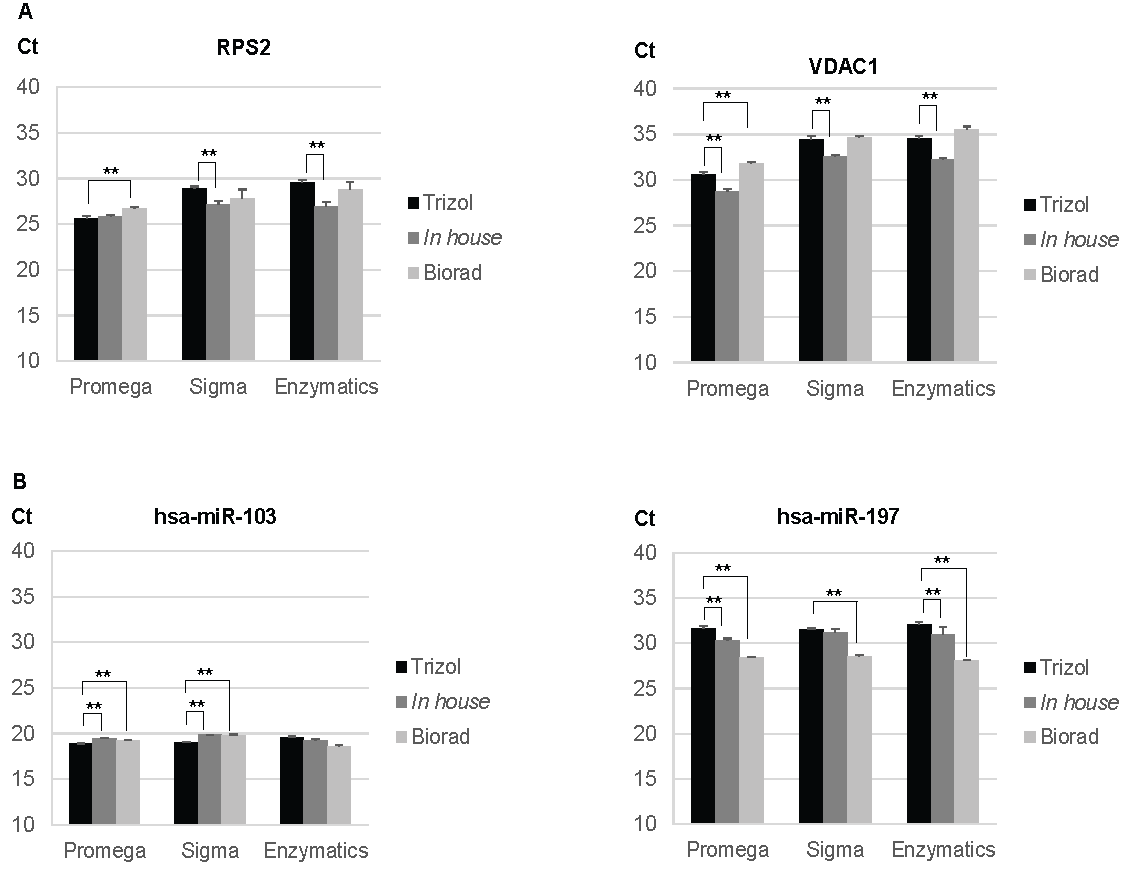

Supplement: Figure S4 — The performances of cell-to-Ct reagents were not dependent on the choice of reverse transcription systems. U251MG cultured in 96-wells at 104 cells per well were directly lysed with Trizol reagent, in-house and Biorad cell-to-Ct reagents. Total RNA samples or cell lysates were then reversed transcribed with various reverse transcription kits (ImProm-II™ Reverse Transcription System, Promega; Enhanced Avian HS RT-PCR Kit, Sigma; M-MuLV Reverse Transcriptase, Enzymatics) according to the manufacturer's instructions. The cDNA samples were amplified and the Ct values determined. Graph bars represented Ct values and the error bars referred to the S.E.M of 3 biological samples. Significant differences in the Ct values between Trizol reagent and cell-to-Ct reagents were calculated using the two tailed student's t-test. *, p<0.01. (TIF) [file pone.0072463.s004.tif]

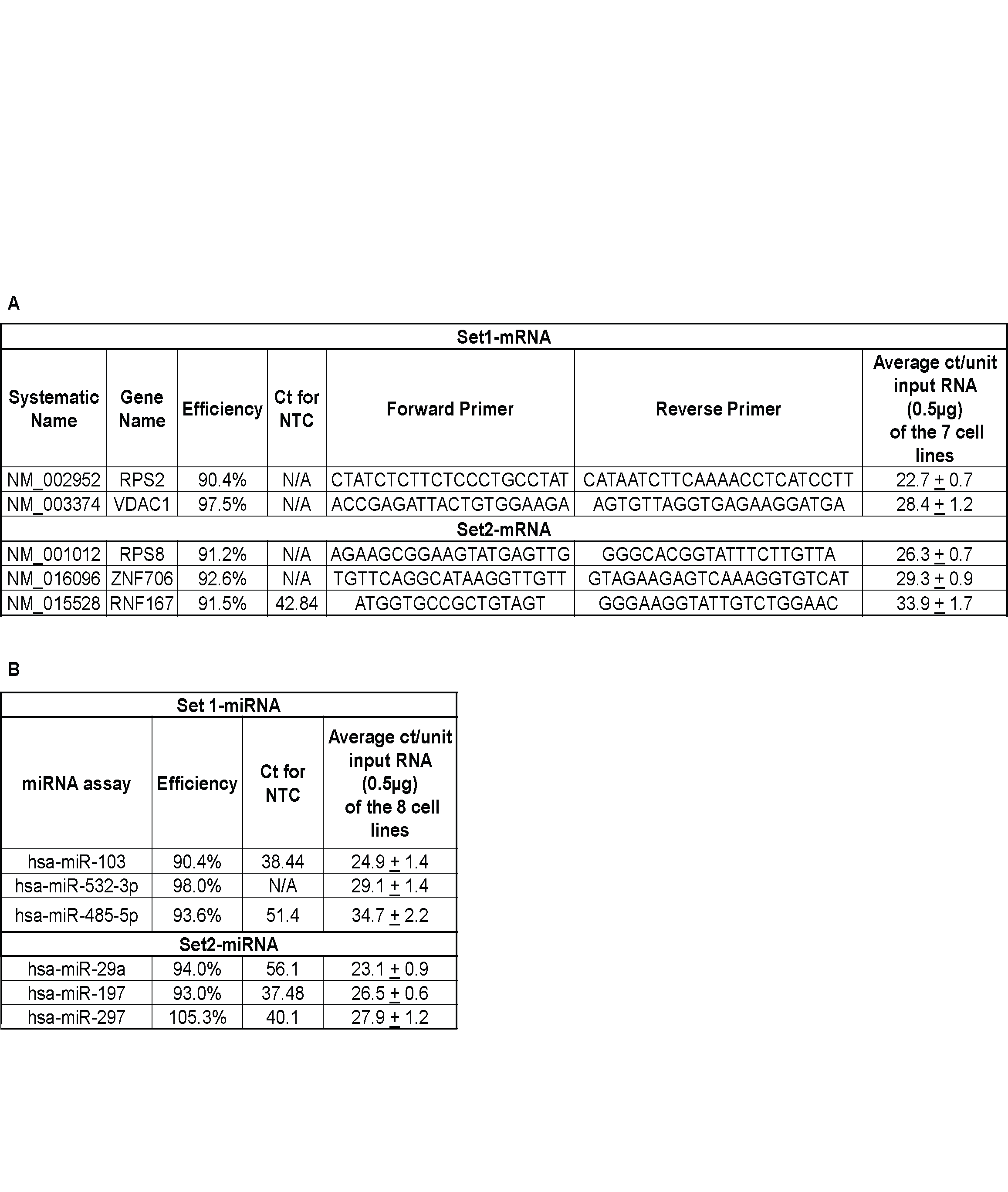

Supplement: Table S1 — RT-qPCR assay design and performance. Target genes were selected from Agilent Whole Human Genome Oligo Microarray and Agilent Human microRNA Microarray V2 data based on the microarray signals. Specific primers for mRNA were designed using Beacon designer. For microRNA detection, miRXES microRNA assays were used. The performance of these assays was validated by testing serial dilutions of cDNA from 786–0 cell line. Efficiencies of amplification were quantified. Average Ct/unit input of RNA, 500ng detected from the total RNA samples of the 8 selected cell lines was reported. (TIF) [file pone.0072463.s005.tif]

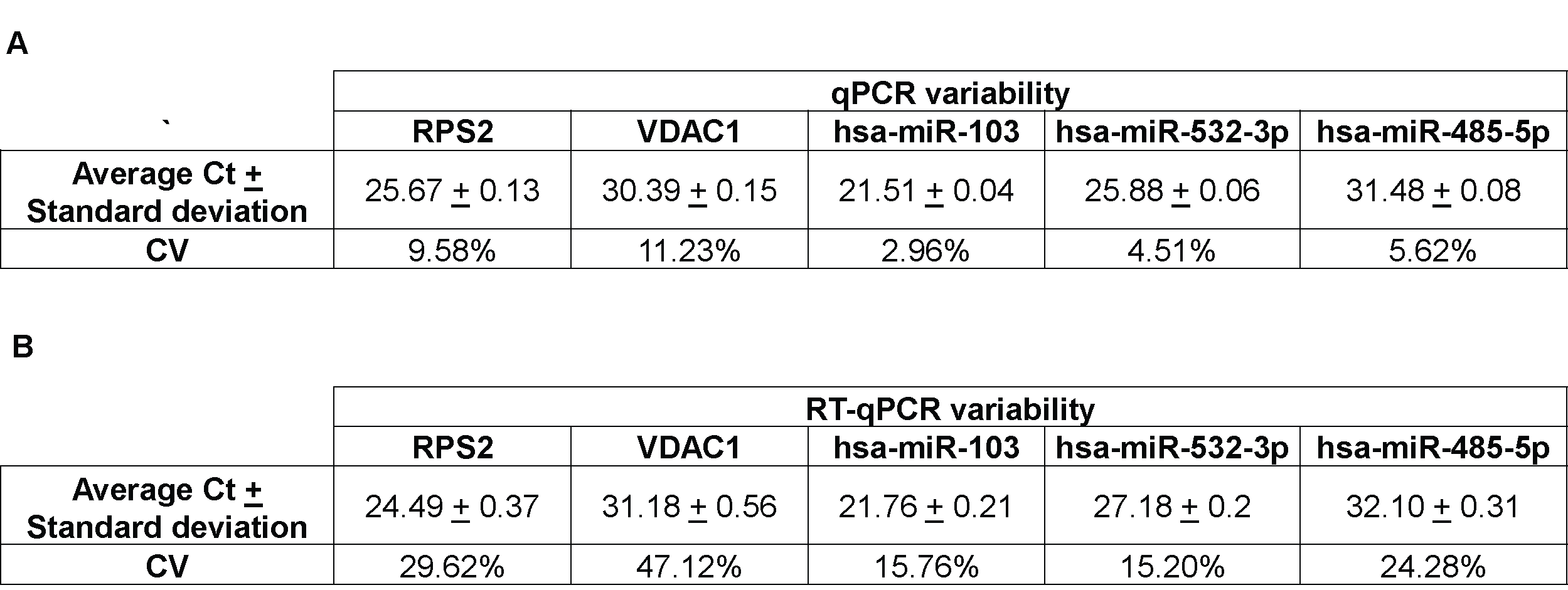

Supplement: Table S2 — Intra assay variation and RT-qPCR variation. (A) Total RNA sample isolated from M14 cells were reversed transcribed. Five replicates of the cDNA sample were amplified with various PCR assays. (B) Five RT reactions were prepared from the RNA sample isolated from M14 cells. The cDNA samples were then amplified by various PCR assays. Table summarizes the average Ct value ± S.D. and CVs of the assays used to measure the selected mRNAs and miRNAs in this study. (TIF) [file pone.0072463.s006.tif]
